# Synergistic Ni–Cu/char bimetallic catalysts for enhanced hydrogen production from corn stover bio-oil *via* steam reforming

**DOI:** 10.1039/d6ra00271d

**Published:** 2026-03-19

**Authors:** Surachai Wongcharee, Nopparat Suriyachai, Torpong Kreetachat, Methawee Nukunudompanich, Supachai Jadsadajerm, Saksit Imman

**Affiliations:** a Department of Environmental Engineering, Faculty of Engineering, Mahasarakham University Mahasarak-ham Thailand; b School of Energy and Environment, University of Phayao Phayao 56000 Thailand; c Integrated Biorefinery Excellence Center (IBC), School of Energy and Environment, University of Phayao Phayao Thailand saksit.im@up.ac.th; d BIOTEC-JGSEE Integrative Biorefinery Laboratory, National Center for Genetic Engineering and Biotech-nology Innovation Cluster 2 Building Pathumthani Thailand; e Department of Industrial Engineering, School of Engineering, King Mongkut's Institute of Technology Ladkrabang Lat Krabang Bangkok 10520 Thailand; f Department of Industrial Chemistry, Faculty of Applied Science, King Mongkut's University of Technology North Bangkok Bangkok 10800 Thailand

## Abstract

Catalytic steam reforming of biomass-derived bio-oil offers a promising route for renewable hydrogen production, yet catalyst deactivation and coke formation limit its practical application, particularly for complex whole bio-oils. Herein, hydrogen production from corn stover-derived whole bio-oil was investigated *via* an integrated fast pyrolysis-steam reforming process using char-supported Ni–Cu bimetallic catalysts. The optimized Ni–Cu composition exhibited enhanced hydrogen yield (∼53%) and feedstock conversion (∼78%), with low carbon deposition compared to monometallic counterparts. Elevated reforming temperatures promoted hydrocarbon cracking and suppressed coke formation. Long-term stability tests demonstrated sustained catalytic performance under steam oxygen reforming conditions. Structural characterization confirmed uniform metal dispersion and preserved catalyst porosity after reaction. The improved performance is attributed to the synergistic interaction between Ni, facilitating C–C bond cleavage, and Cu, enhancing water–gas shift activity and mitigating carbon deposition. These findings highlight the potential of char-supported Ni–Cu catalysts as a robust and coke-resistant system for scalable hydrogen production from real biomass-derived bio-oil.

## Abstract 2

Catalytic steam reforming of biomass-derived bio-oil offers a promising route for renewable hydrogen production, yet catalyst deactivation and coke formation limit its practical application, particularly for complex whole bio-oils.

## Graphical abstract

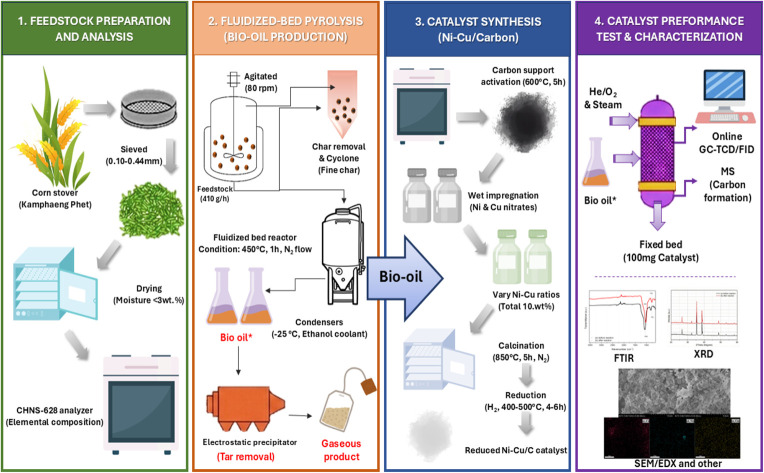

## 1. Introduction

At present, the world is continuously advancing with diverse modern technologies driven by the increasing demands of society, alongside the growing need for energy. From the past to today, the primary energy source has largely been finite fossil fuels. This reliance has contributed to global warming, which is intensifying due to the emission of various greenhouse gases such as NO_*x*_ and So_*x*_.^1^ Such emissions may lead to catastrophic consequences, raising current concerns regarding the depletion of fossil fuels and the exacerbation of global warming for life on Earth.^2^ For these reasons, the urgent development of sustainable solutions is imperative to prevent the global temperature from exceeding critical thresholds. Among the alternatives, renewable energy is expected to play a central role in achieving net-zero greenhouse gas emissions.^3^

In general, renewable resources provide sustainable and inexhaustible energy options. Among them, biomass is particularly significant due to its potential to produce both high-value chemicals and essential energy carriers such as hydrogen. Hydrogen (H_2_) production represents a highly attractive alternative, as it offers a low-cost energy source that can be generated in substantial quantities, making it economically viable for industrial applications.^4^ At the same time, hydrogen can be derived from diverse biomass resources, including agricultural residues, crop by-products, organic waste from livestock farms, and community-generated organic waste.^5^ Currently, hydrogen energy is utilized as a key feedstock in various industries, including rocket fuels, hydrogen-powered vehicles, and beyond.^6^ Therefore, within the current context of the energy transition and global efforts to mitigate greenhouse gas emissions, the utilization of biomass for hydrogen production as a renewable energy pathway has gained increasing attention.^7^

Hydrogen can be produced through biological and thermochemical processes at high temperatures, as well as from fossil fuels *via* steam methane reforming or pyrolysis. In addition, water electrolysis is a well-established route for hydrogen generation. Currently, fast pyrolysis coupled with steam reforming, a two-step thermochemical process for producing hydrogen-rich syngas from biomass, offers significant advantages over biological pathways, providing higher yields and greater flexibility depending on the type of biomass feedstock.^8^ Integrated pyrolysis and catalytic steam reforming (CSR) has been proposed as a promising two-step pathway for hydrogen production from biomass-derived bio-oil.^9^ Fast pyrolysis of biomass under oxygen-free conditions at moderate temperatures yields a high proportion of bio-oil, which serves as an effective feedstock for catalytic steam reforming. Conducted at elevated temperatures (500–900 °C), this process enables high hydrogen yields, making CSR a potential sustainable route for biohydrogen production.

Catalysts for the steam reforming of bio-oil are generally metal-based and supported on metal oxides. Among them, nickel (Ni)-based catalysts are the most widely studied and applied due to their high activity in steam reforming of hydrocarbons, including bio-oil. Their superior performance arises from the high efficiency in cleaving C–C and C–H bonds, combined with low cost and wide availability. However, the type and design of catalysts play a crucial role in determining hydrogen yield and in minimizing undesirable by-products such as coke.^10^ investigated the enhanced stability of Ni/Al_2_O_3_ catalysts promoted with MgO for bio-oil steam reforming. They further examined Ni-based catalysts promoted with various oxides such as Mg, La, and Ce. The results demonstrated that Ni/MgO–Al_2_O_3_ exhibited significantly higher stability during a 12 h time-on-stream test. The incorporation of MgO increased the basicity of the catalyst support, which facilitated steam adsorption and activation, thereby promoting the gasification of carbonaceous intermediates and significantly reducing coke formation compared to unpromoted Ni/Al_2_O_3_. Similarly,^11^ studied bimetallic Ni–Cu catalysts supported on ZrO_2_ for the steam reforming of acetic acid. Their findings revealed that Ni–Cu/ZrO_2_ with a Ni/Cu ratio of 3 : 1 outperformed monometallic Ni catalysts, not only achieving higher hydrogen yields but also demonstrating greater stability over 24 h. The presence of Cu promoted the water–gas shift (WGS) reaction, suppressed coke deposition, and improved hydrogen selectivity. Nevertheless, rapid catalyst deactivation due to coke deposition and sintering remains a major challenge, resulting in reduced hydrogen yield and gas efficiency. To address these issues, the use of promoters and the modification of catalyst support have been considered effective strategies.

This study aims to investigate the development of catalysts for whole bio-oil derived from corn stover (BO-CS) under pyrolysis-catalytic steam reforming, focusing on gas yield enhancement and coke reduction under optimized conditions. Therefore, the development and optimization of catalysts are essential for ensuring both the economic sustainability and the scalability of biohydrogen production *via* steam reforming of bio-oil.

## 2. Materials and methods

**Fig. 1:**
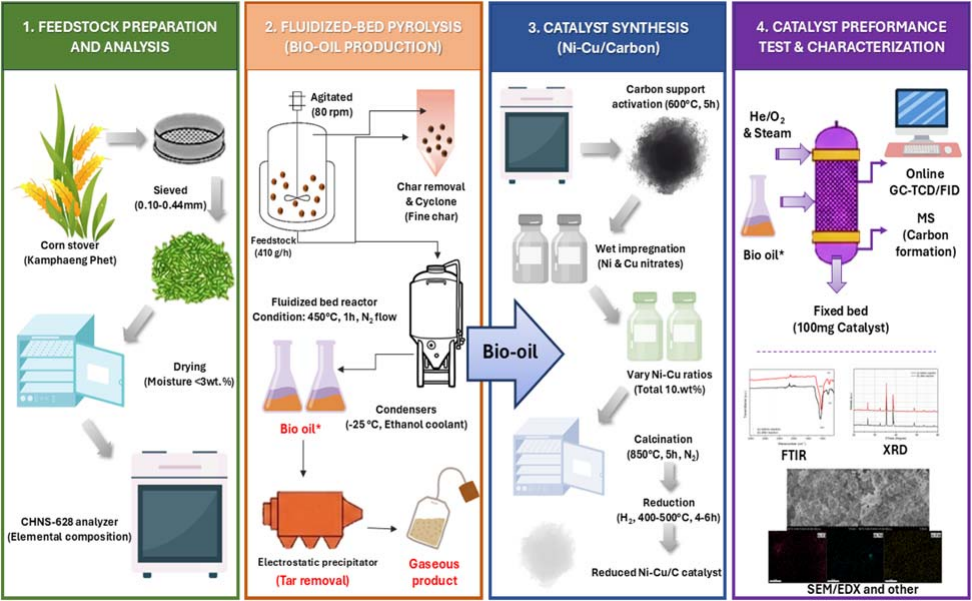
Process flow diagram of corn stover conversion to hydrogen *via* pyrolysis-catalytic reforming.

The experimental materials, catalyst synthesis protocols, reactor operation conditions, and analytical techniques (Fig. 1) applied in this study are described in detail in the following subsections.

### 2.1 Feedstock

Corn stover used in this study was sourced from local agricultural fields in Kamphaeng Phet Province, Thailand. Prior to use, the biomass was mechanically sieved to obtain a particle size fraction of 0.13–0.44 mm and then oven-dried at 90 °C for 24 h to remove residual moisture. After drying, the moisture content of the prepared feedstock was reduced to below 3 wt%, ensuring stable and reproducible thermochemical conversion. The physicochemical properties of the corn stover were characterized through proximate and ultimate analyses, along with compositional determination of its main structural components, including cellulose, hemicellulose, lignin, ash, and extractives. Elemental analysis was conducted using a CHNS-628 analyzer (LECO, USA). Prior to measurement, samples were vacuum dried at 60 °C to eliminate adsorbed moisture. Carbon, hydrogen, and nitrogen contents were determined from approximately 0.5 g of sample, while sulfur content was measured separately by high-temperature oxidation at 1350 °C and quantified using an infrared sulfur detector. The resulting compositional data provided the basis for evaluating the suitability of corn stover as a feedstock for pyrolysis and subsequent catalytic steam reforming.

### 2.2 Fluidized-bed pyrolysis reactor

Fast pyrolysis of corn stover was performed prior to catalytic testing using a laboratory-scale fluidized-bed reactor fabricated from SUS 306 stainless steel. The prepared biomass was continuously introduced into the reactor at a feed rate of 410 g h^−1^, while high-purity nitrogen was supplied at a flow rate of 7 L min^−1^ to maintain an oxygen-free atmosphere throughout the process. Mechanical agitation at 80 rpm was applied to enhance particle mixing and heat transfer within the reactor. Pyrolysis experiments were conducted at 450 °C for a duration of 1 h. Both the reactor body and gas inlet lines were electrically heated to ensure stable operating conditions. The reactor system was equipped with a char separation unit and a cyclone separator for the effective removal of fine char particles with sizes ranging from 2 to 10 µm. Condensable pyrolysis vapors were recovered using a series of glass condensers maintained at −25 °C by a recirculating cooling system (RW-2025G, JEIO TECH) employing ethanol as the cooling medium. Vapors that were not condensed were subsequently passed through an electrostatic precipitator to remove residual tar compounds. The remaining non-condensable gases were collected in gas sampling bags for subsequent compositional analysis.^12^ The chemical composition of the condensed bio-oil was characterized both qualitatively and quantitatively using gas chromatography-mass spectrometry (GC-MS, HP 5973 inert) equipped with an HP-5MS capillary column (30 m × 0.25 mm × 0.25 µm). The GC-MS results were further used to calculate the bio-oil yield, which served as the feedstock for subsequent catalytic steam reforming experiments.

### 2.3 Catalyst preparation

The carbon support was initially prepared using a stainless-steel tubular reactor. Commercial activated carbon (≥99% purity, particle size 100–200 mesh, BET surface area ∼900 m^2^ g^−1^, Sigma-Aldrich) was used as the carbon precursor without further purification. A predetermined amount of carbon material was placed on a sample holder inside the reactor and heated to 550 °C at a controlled rate of 10 °C min^−1^ under a continuous flow (100 mL min^−1^) of high-purity nitrogen (N_2_, 99.9%, Linde). This treatment was maintained for 2 h to stabilize the carbon structure, after which the reactor was allowed to cool naturally to ambient temperature under an inert atmosphere. The obtained carbon was then subjected to an activation step at 600 °C for 5 h to enhance its surface properties prior to metal impregnation.

Bimetallic Ni–Cu catalysts with different Ni to Cu ratios (10 : 0, 8 : 2, 6 : 4, 4 : 6, and 0 : 10) were synthesized by a wet impregnation method, while maintaining a constant total metal loading of 10 wt% with respect to the carbon support. Stoichiometric amounts of nickel nitrate hexahydrate (Ni (NO_3_)_2_·6H_2_O) and copper nitrate trihydrate (Cu (NO_3_)_2_·3H_2_O) (Ni (NO_3_)_2_·6H_2_O, ≥98% purity, Sigma-Aldrich; Cu (NO_3_)_2_·3H_2_O, ≥99% purity, Sigma-Aldrich) were dissolved in deionized water (resistivity 18.2 M Ω·cm) and gradually introduced onto the carbon support with continuous mixing to promote uniform metal distribution. The impregnated samples were aged at room temperature for 12 h to ensure proper metal dispersion and subsequently dried at 110 °C overnight (12 h).

Following drying, the samples were calcined at 850 °C for 7 h under a nitrogen atmosphere (N_2_, 99.9% purity, 100 mL min^−1^) to decompose the metal precursors and stabilize the supported metal phases. Prior to catalytic evaluation, the catalysts were reduced in a hydrogen stream (H_2_, 99.9% purity, 50 mL min^−1^) at 400 °C for 4 h using a heating rate of 2 °C min^−1^. After the reduction step, the catalysts were cooled under an inert atmosphere and stored in airtight containers until further use. For comparison purposes, monometallic Ni/C (10 wt% Ni) and Cu/C (10 wt% Cu) catalysts were prepared following the same impregnation, calcination, and reduction procedures and were used as authentic reference samples to evaluate the synergistic effect between Ni and Cu in the bimetallic catalysts.

### 2.4 Evaluation of catalytic performance

Catalytic activity experiments were conducted in a fixed-bed reactor housed within an electrically heated tubular furnace and operated at atmospheric pressure. During each experiment, helium was used as the carrier gas, while oxygen was introduced when required; gas flow rates were precisely regulated using mass flow controllers. Bio oil was supplied to the system through a saturator unit, and steam was delivered using a heated syringe pump. The steam was subsequently vaporized and mixed with the reactant gases using a custom-designed quartz vaporizer mixer. To prevent condensation of tar or heavy hydrocarbons, all gas transfer lines were maintained at 250 °C. Reactor temperature was controlled by a programmable temperature controller to ensure stable operating conditions. Each catalytic test was performed using 100 mg of catalyst, and the total gas flow rate was fixed at 100 cm^3^ min^−1^. Prior to activity measurements, the catalyst was reduced *in situ* under a flow of 10% H_2_ in Helium at 500 °C for 6 h to generate the active metallic phase. The composition of gaseous reaction products was analyzed online using a Shimadzu GC-14B gas chromatograph equipped with both a thermal conductivity detector (TCD) and a flame ionization detector (FID). A mass spectrometer was additionally employed to support carbon formation analysis. Catalytic performance was evaluated in terms of bio-oil conversion and product distribution, including CO, CO^2^, CH^4^, and C^2+^ hydrocarbons. Product distributions were determined based on carbon balance and expressed as the molar fraction of each product relative to the total moles of hydrocarbon converted. Hydrogen yield was calculated from a hydrogen balance and defined as the molar fraction of hydrogen produced relative to the total hydrogen in the reactant feed. Following completion of the catalytic tests, carbon deposition on spent catalysts was quantified by temperature-programmed oxidation (TPO). This analysis was carried out by exposing the catalysts to a gas mixture containing 10% O_2_ in Helium at temperatures up to 1000 °C. The amounts of carbon deposited were determined from the measured concentrations of CO and CO_2_ evolved during oxidation.

### 2.5 Catalyst characterization

The physicochemical properties of the prepared catalysts were characterized using a combination of spectroscopic, microscopic, and structural analysis techniques. Functional groups present on the catalyst surfaces were identified by Fourier transform infrared spectroscopy (FT-IR) using a PerkinElmer System 2000 spectrometer (Waltham, USA). Spectra were collected over a wavenumber range of 400–4000 cm^−1^. X-ray photoelectron spectroscopy (XPS) analysis was performed using a thermo scientific K-Alpha spectrometer equipped with Al Kα radiation (1486.6 eV). The binding energies were calibrated using C 1s at 284.8 eV as reference.

The surface morphology and microstructural features of the catalysts were examined using scanning electron microscopy (SEM, JEOL JSM-6610 LV) operated at an accelerating voltage of 20 kV. Carbon deposition on spent catalysts was quantified by temperature-programmed oxidation (TPO), which was conducted under a flowing gas mixture of 10% O_2_ in He up to 1000 °C. The evolution of CO and CO_2_ during oxidation was continuously monitored using a mass spectrometer to determine the amount of deposited carbon.

Crystalline phases and structural properties of the catalysts were analyzed by X-ray diffraction (XRD) using a Philips X'Pert diffractometer equipped with Cu Kα radiation (*λ* = 1.5406 Å), operated at 40 kV and 30 mA. Diffraction patterns were recorded at ambient temperature over a 2*θ* range of 5°–80° with a step size of 0.02° min^−1^. The crystallinity index (CrI) was calculated using a standard method, as defined by the following equation.1
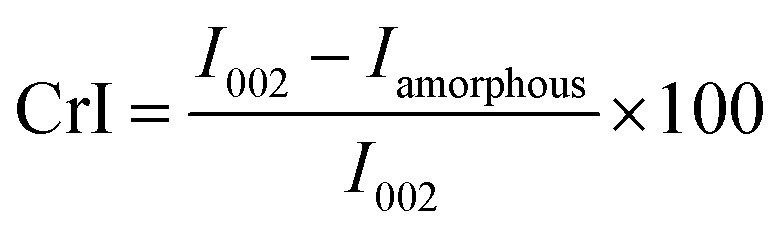


## 3. Results and discussion

### 3.1 Chemical composition and ultimate analysis of corn stover

**Fig. 2:**
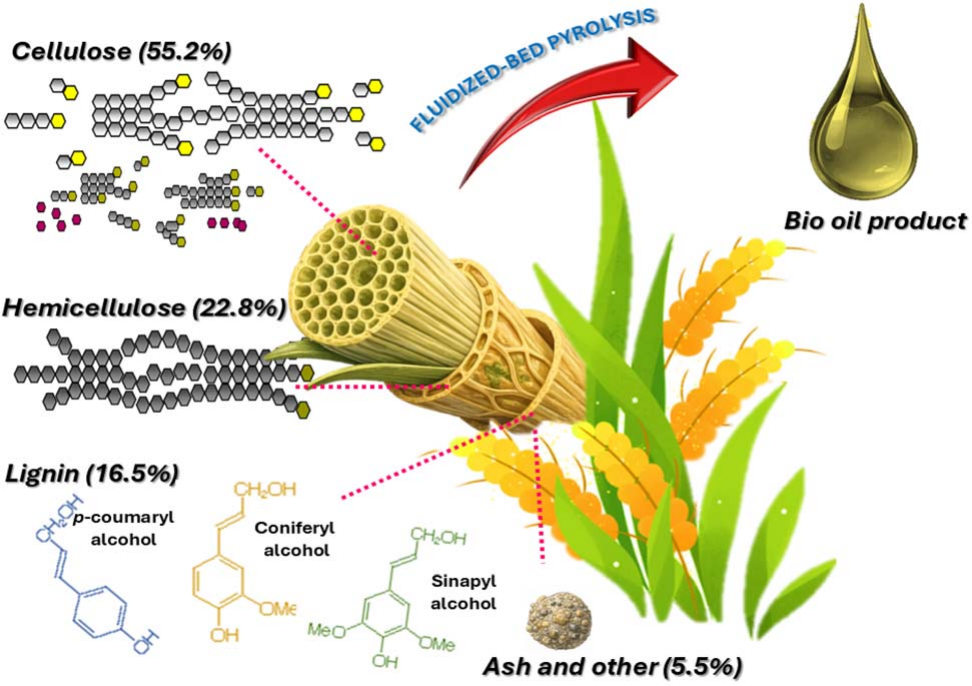
Schematic illustration of corn stover composition and its transformation into oxygenated bio-oil during pyrolysis.

The compositional properties of raw corn stover were analyzed prior to fast pyrolysis, and the results are presented in Table 1 and Fig. 2.

Characteristics of raw corn stover

**Table:** 

Chemical composition	(wt% on dry basis)
Cellulose	55.2
Hemicellulose	22.8
Lignin	16.5
Ash	4.6
Another extractive	0.9

**Table:** 

Ultimate analysis	(wt%)
Carbon	49
Hydrogen	5.21
Nitrogen	1.5
Oxygen	44.08
Sulfur	<0.2

The biomass consisted predominantly of carbohydrate polymers, with cellulose accounting for 55.2 wt% and hemicellulose for 22.8 wt%, giving a combined polysaccharide content of nearly 78 wt%. This value is comparatively higher than those reported for several other agricultural residues, such as rice straw (cellulose 32–40 wt%, hemicellulose 20–25 wt%) and wheat straw (cellulose 35–45 wt%, hemicellulose 25–30 wt%), indicating a strong potential for volatile release and liquid product formation during fast pyrolysis. Lignin constituted 16.5 wt% of the corn stover, which is lower than that typically observed in woody biomass (20–30 wt%) but comparable to residues such as sugarcane bagasse (18–22 wt%). This moderate lignin content contributes to char formation while maintaining favorable bio-oil yields. The ash content was relatively low at 4.6 wt%, notably lower than that of rice husk (15–20 wt%) and comparable to corn cob and wheat straw, suggesting reduced risks of slagging, catalyst poisoning, and inorganic interference during thermochemical conversion.

Ultimate analysis revealed that carbon was the dominant element (49.0 wt%), followed by oxygen (44.08 wt%) and hydrogen (5.21 wt%), with nitrogen (1.5 wt%) and sulfur (<0.2 wt%) present only in minor amounts. The carbon content of corn stover is similar to that of other lignocellulosic residues such as wheat straw (47–49 wt%) and sugarcane bagasse (45–48 wt%), while its relatively high oxygen content reflects the abundance of oxygenated functional groups inherent to biomass materials. Although this high oxygen fraction implies a lower heating value compared to fossil-derived feedstocks, it favors the formation of oxygen-rich compounds including acids, aldehydes, ketones, and phenolics during pyrolysis. These oxygenated species are highly reactive in downstream catalytic steam reforming, where they strongly influence hydrogen production efficiency, syngas composition, and coke formation tendencies. Consequently, the compositional profile of corn stover highlights its suitability as a feedstock for integrated pyrolysis-steam reforming routes aimed at hydrogen-rich gas production.

### 3.2 The principal compounds produced during fast pyrolysis of corn stover

Prior to catalytic steam reforming, raw corn stover was thermochemically converted into bio-oil under optimized fast pyrolysis conditions. The resulting product distribution is summarized in Table 2. When pyrolysis was carried out at 500 °C for 1 h, bio-oil was obtained with a yield of 45.91 wt% based on differential calculation, while direct gravimetric measurement yielded 40.43 wt%. In addition to liquid products, char and non-condensable gas fractions accounted for 24.88 wt% and 29.21 wt%, respectively.

**Table 2:** Product yields obtained from fast pyrolysis of corn stover^a^

Product fraction	Yield (wt%)
Oil yield by difference	45.91
Char	24.88
Gas	29.21
Oil yield by direct measurement	40.43
Bio-oil loss	5.48

a Calculate by wt% on dry basis.

The difference between the calculated and directly measured bio-oil yields, corresponding to a loss fraction of 5.48 wt%, can be attributed primarily to incomplete condensation and the escape of light volatile compounds during the collection process. The relatively high liquid yield obtained under these conditions indicates that the selected pyrolysis parameters were effective in promoting the thermal decomposition of corn stover and facilitating the conversion of volatile intermediates into condensable bio-oil. Such a product distribution is favorable for subsequent catalytic steam reforming, as the bio-oil fraction serves as a suitable feedstock for hydrogen production.

These results are consistent with previous reports. In the fast pyrolysis of corn stover, bio-oil yields of 40–50% have typically been observed under operating temperatures of 450–550 °C.^13^;^14^ The observed char yield of up to 24.88% can be attributed to the high aromatic and thermally stable lignin content of corn stover.^15^ The gas fraction, meanwhile, is primarily generated through the secondary cracking of volatile intermediates and the decomposition of cellulose and hemicellulose, leading to the formation of CO, CO_2_, and light hydrocarbons.^16^ Similarly,^17^ and^18^ reported that optimizing reactor temperature and vapor residence time can significantly increase the bio-oil yield while maintaining balanced proportions of gas and char, thereby enhancing the efficiency of downstream processing.

Eqn (2)–(9) presents proposed reaction mechanism for steam reforming over Ni–Cu/char catalysts. The steam reforming of bio-oil over Ni–Cu/char catalysts is proposed to proceed through a sequence of adsorption, reforming, shift, and regeneration steps. Initially, bio-oil oxygenates and aromatic compounds, represented by bio-oil (C_10_H_8_), are adsorbed and activated on metallic Ni sites through strong π-metal interactions, forming surface-bound species that facilitate C–C and C–H bond activation. In the presence of steam, these activated hydrocarbons undergo Ni-dominated primary steam reforming, leading to extensive hydrocarbon cracking and the formation of CO and H_2_ as the main intermediate products. The CO generated during this step is subsequently converted *via* the water-gas shift reaction, which is promoted by Cu sites, producing additional hydrogen while reducing CO concentration. When steam reforming and complete water–gas shift reactions are combined, the overall ideal conversion corresponds to the stoichiometric transformation of C_10_H_8_ and steam into CO_2_ and H_2_, with a maximum theoretical hydrogen yield of 24 mol H_2_ per mol of naphthalene. Under conditions of insufficient steam or partial catalyst deactivation, undesired side reactions may occur, including aromatic condensation and CO disproportionation (Boudouard reaction), resulting in the formation of solid carbon (coke), which is typically accelerated on Ni-rich surfaces. However, the presence of Cu weakens carbon metal interactions and facilitates coke suppression. Deposited carbon can be removed through steam gasification to form CO and H_2_, followed by further conversion *via* the water–gas shift reaction. This synergistic interaction between Ni and Cu explains the enhanced hydrogen yield, reduced carbon deposition, and improved catalytic stability observed for the Ni–Cu/char catalyst, particularly at an optimized Ni–Cu ratio.

Adsorption and activation on metal sites2
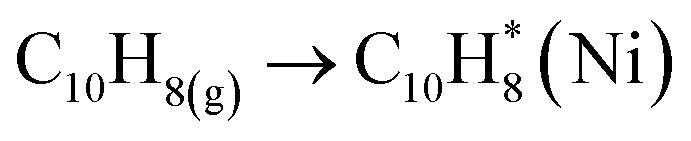


Primary steam reforming (Ni-dominated)3
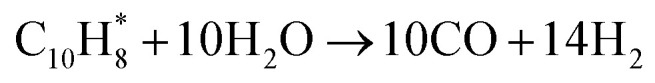


Water–gas shift reaction (Cu-promoted)

4 CO + H_2_O ↔ CO_2_ + H_2_

Overall ideal reforming reaction

5 C_10_H_8_ + 20H_2_O → 10CO_2_ + 24H_2_

Coke formation pathways (undesired)

6 C_10_H_8_ → C_(s)_ + H_2_

7 2CO → CO_2_ + C_(s)_

Coke removal *via* steam gasification

8 C_(s)_ + H_2_O → CO + H_2_

9 CO + H_2_O → CO_2_ + H_2_

### 3.3 Effect of Ni–Cu/char catalysts with different metal ratios on hydrogen yield and conversion

The influence of Ni–Cu/char catalysts with varying Ni-to-Cu ratios on hydrogen production and feedstock conversion during the steam reforming of corn stover bio-oil was systematically evaluated. Experiments were carried out at 800 °C under steam reforming conditions for reaction times of 1 h and 24 h, and the results are presented in Fig. 1 and 2. Comparative performance was assessed for the non-catalytic case, char alone, and Ni–Cu/char catalysts with Ni–Cu ratios of 10 : 0, 8 : 2, 6 : 4, 4 : 6, and 0 : 10.

In the absence of a catalyst, both hydrogen yield and conversion were notably low, highlighting the limited extent of thermal reforming under these conditions. The introduction of char led to a moderate enhancement in performance, whereas the incorporation of nickel-based catalysts resulted in a substantial improvement in hydrogen production. For the monometallic Ni/char catalyst (10 : 0), the hydrogen yield increased to 39.5% after 1 h of reaction, confirming the strong reforming activity of Ni for C–C and C–H bond cleavage.

Among all tested catalysts, the Ni–Cu/char (8 : 2) sample exhibited the highest catalytic performance, achieving a hydrogen yield of 52.9% and a feedstock conversion of 77.7% after 1 h at 800 °C. This superior activity is attributed to the synergistic interaction between Ni and Cu, which enhances reforming reactions while mitigating catalyst deactivation. In contrast, further increases in Cu content led to a gradual decline in catalytic efficiency. Specifically, Ni–Cu/char catalysts with ratios of 6 : 4 and 4 : 6 exhibited reduced hydrogen yields of 46.7% and 41.1%, respectively, accompanied by lower conversion values of 70.1% and 66.5% (Table 3). The Cu-rich catalyst (0 : 10) showed the poorest performance among the metal-loaded samples, indicating that Cu alone provides limited reforming activity. Overall, these results demonstrate that an optimal Ni–Cu ratio is essential for maximizing hydrogen yield and conversion. Excessive Cu content suppresses reforming efficiency, while a balanced Ni–Cu composition particularly at a ratio of 8 : 2 offers the most effective catalytic performance for the steam reforming of corn stover bio-oil.

**Table 3:** Hydrogen yield from Ni–Cu/char catalysts with varying Ni–Cu/ratios under pyrolysis steam reforming conditions

Catalyst ratio	Conversion		H_2_ yield	
	Conversion (1 h)	Conversion (24 h)	H_2_ yield (1 h)	H_2_ yield (24 h)
Non catalyst	10.2	8.1	6.1	2.34
Char	38.9	32.4	18.2	13.5
Ni–Cu/char (10 : 0)	65.3	56.8	39.5	28.7
Ni–Cu/char (8 : 2)	77.7	63.8	52.9	39.0
Ni–Cu/char (6 : 4)	70.1	56.2	46.7	35.2
Ni–Cu/char (4 : 6)	66.5	52.1	41.1	30.8
Ni–Cu/char (0 : 10)	59.6	44.0	36.7	19.1

Based on stoichiometric analysis, the maximum theoretical hydrogen yield for steam reforming of bio-oil (C_10_H_8_), used as a model compound for bio-oil, is 24 mol H_2_ per mol of feed under complete reforming and water–gas shift conditions. The experimentally observed hydrogen yield of 52.9% over the Ni–Cu/char (8 : 2) catalyst therefore corresponds to approximately 12.7 mol H_2_ per mol of naphthalene, indicating that more than half of the stoichiometric hydrogen potential was achieved. This result demonstrates the effectiveness of the Ni–Cu synergy in approaching ideal reforming behavior while limiting catalyst deactivation.

It is evident that the presence of nickel substantially enhanced both conversion and hydrogen yield compared with Cu- or char-supported catalysts. The maximum catalytic activity was obtained at a Ni–Cu ratio of 8 : 2, which produced the highest hydrogen yield and conversion after 1 h while maintaining relatively low carbon deposition (4.02 mmol g_cat_^−1^). Further increasing the Cu content beyond this ratio led to a continuous decline in conversion and hydrogen yield, with the lowest performance observed for Cu alone, indicating reduced reforming capability for the Cu-rich catalyst. Conversely, higher Cu ratios promoted carbon formation (up to 15.9 mmol g_cat_^−1^), which negatively affected hydrogen yield and conversion by blocking active sites on the catalyst surface. Nevertheless, the incorporation of Cu effectively suppressed coke formation compared with the monometallic Ni catalyst, demonstrating a synergistic interaction between Ni and Cu that improved catalyst stability and resistance to carbon deposition over both 1 h and 24 h reaction durations.

### 3.4 Impact of reaction time on hydrogen yield during steam reforming of BO-CS under a fixed H_2_O/C inlet ratio

**Fig. 3:**
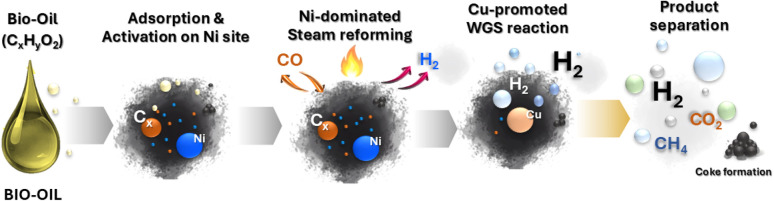
Proposed reaction pathway for steam reforming of whole bio-oil (C_*x*_H_*y*_O_2_) over Ni–Cu/char catalysts, illustrating oxygenate activation and reforming on Ni sites, CO conversion *via* the Cu-promoted water-gas shift reaction.

The effect of reaction duration under a fixed inlet H_2_O/C ratio on hydrogen production during the steam reforming of BO-CS was investigated. As shown in Fig. 3, a comparison was made among the catalysts char, Ni/char, Cu/char, and Ni–Cu/char (8 : 2) in the steam reforming process of BO-CS at 800 °C under a constant H_2_O/C ratio. Hydrogen yields were measured over a period of 5 h, with data collected at the time intervals of 0, 0.5, 1, 1.5, 2, 2.5, 3, 3.5, 4, 4.5, and 5 h, respectively. The results revealed that the hydrogen yield of all catalysts exhibited an increasing trend with reaction time, showing a maximum hydrogen production between 1–1.5 h for all catalysts. Notably, when comparing catalysts, the Ni–Cu/char catalyst with a ratio of 8 : 2 exhibited the highest and most stable hydrogen yield throughout the entire reaction period, reaching approximately 53.4% at 1 h and decreasing slightly to 40.57% after 5 h, which was significantly higher than those obtained using other catalysts. This catalyst demonstrated a continuous increase in hydrogen yield, with a distinct peak observed between 1–1.5 h, followed by a gradual decline thereafter. In comparison, the hydrogen yield under the condition using only char remained consistently low throughout the reaction, starting at 9.3% and increasing only slightly with time, indicating its poor catalytic potential. For Ni/char (10 : 0) and Cu/char catalysts, the hydrogen yields increased moderately, with the Ni-char catalyst producing higher hydrogen yields than the Cu-char catalyst.

However, both catalysts still lagged the Ni–Cu/char (8 : 2) catalyst, showing a decrease in hydrogen yield after 5 h. The increasing trend of hydrogen yield with reaction time corresponded well with the catalytic behavior, as more hydrocarbon cracking occurred as the reaction progressed, leading to higher hydrogen release. The superior performance of the Ni–Cu/char (8 : 2) catalyst compared to the monometallic Ni/char and Cu/char catalysts was attributed to the synergistic interaction between Ni and Cu at the 8 : 2 ratio. The presence of Cu enhanced the cracking and reforming processes at 800 °C and helped reduce coke deposition, which is a major factor contributing to the decline in hydrogen yield. The Ni–Cu/char (8 : 2) catalyst also exhibited the most effective catalytic behavior over time, producing higher hydrogen yields more rapidly and maintaining its activity longer than other catalysts. In contrast, the char-only catalyst produced the lowest hydrogen yield throughout the entire reaction period, indicating limited catalytic activity and highlighting the necessity of metal-supported catalysts to improve the process efficiency. Both Ni/char and Cu/char catalysts showed moderate performance; however, neither achieved hydrogen yields comparable to those of the Ni–Cu/char (8 : 2) catalyst.

Previous studies have reported that the combined presence of Ni and Cu creates a more favorable catalytic environment for hydrogen production, as Ni facilitates the cleavage of C–C and C–H bonds, while Cu promotes the gasification of residual carbon and mitigates coke formation.^19,20^ Therefore, under an inlet H_2_O/C ratio at a reaction duration of 1 h, the Ni–Cu/char (8 : 2) catalyst exhibited the highest hydrogen production efficiency from BO-CS, demonstrating superior catalytic stability and performance throughout the 0–5 h reaction period.

### 3.5 Effect of reaction temperature on hydrogen production, conversion, and product distribution in the catalytic steam reforming of BO-CS using Ni–Cu/char (8 : 2) catalyst

**Fig. 4:**
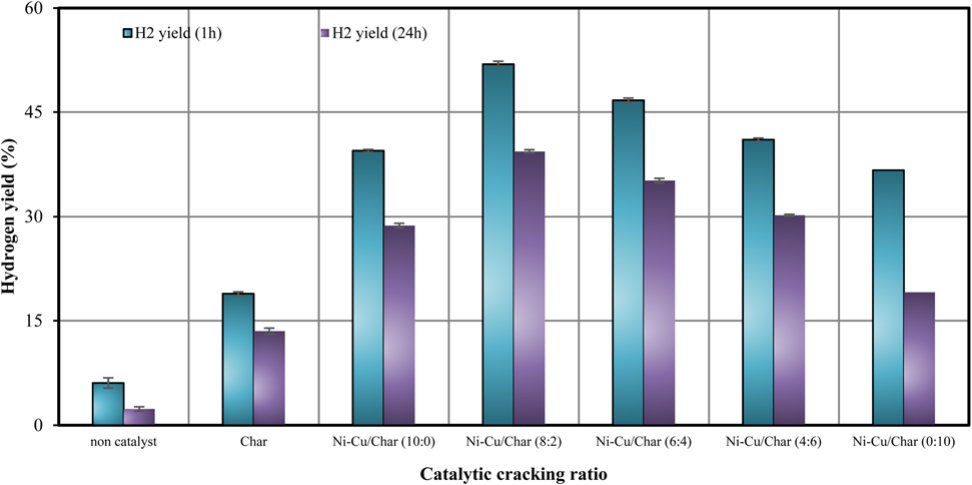
Hydrogen yield from Ni–Cu/char catalysts with varying Ni–Cu/ratios under pyrolysis-steam reforming conditions.

The effect of temperature in the range of 600–800 °C on the product distribution, including CO, CO_2_, CH_4_, and C_2_^+^, hydrogen yield (%), feedstock conversion (%), and carbon formation (mmol g_cat_^−1^), under the catalytic steam reforming process of BO-CS using the Ni–Cu/char (8 : 2) catalyst, was investigated as shown in Fig. 4. The results revealed a strong correlation between increasing temperature and both hydrogen yield and feedstock conversion. The experimental results showed that the hydrogen yield increased continuously with temperature, starting from 56.2% at 600 °C. Similarly, the maximum BO-CS conversion reached 75.1% at 800 °C, while the hydrogen conversion increased from 68.4% to 80.9%, respectively. It can be observed that thermodynamic enhancement at higher temperatures led to more efficient decomposition and reforming of oxygenated compounds present in the bio-oil.

Furthermore, Fig. 4 also shows that the composition of the product gases varied significantly with temperature. The CO_2_ production increased markedly from approximately 24.4% at 600 °C to 52.6% at 800 °C, while the CO concentration moderately decreased from 50.1% to about 40.5%. The inverse relationship between CO and CO_2_ concentrations indicates a shift in the water–gas shift equilibrium toward CO_2_ formation, thereby promoting higher hydrogen generation at elevated temperatures. In contrast, the concentrations of CH_4_ and heavier hydrocarbons (C_2_^+^) continuously decreased with increasing temperature. The CH_4_ content decreased from 16% to less than 2.7%, while the C_2_^+^ compounds dropped from approximately 2.8% to 0%. The higher temperature promoted thermal cracking and reforming of hydrocarbons into syngas components instead. In addition, carbon deposition at lower temperatures exhibited a higher accumulation of 6.7 mmol g_cat_^−1^. Similarly, the amount of carbon formation decreased from approximately 6.7 mmol g_cat_^−1^ at 600 °C to 4.1 mmol g_cat_^−1^ at 800 °C. The reduction of coke deposition at higher temperatures indicates the ability of the Ni–Cu/char (8 : 2) catalyst to suppress carbon accumulation. The decreased carbon formation also reflects the improved gas-phase reforming and gasification of intermediate carbonaceous species at higher temperatures. Therefore, the Ni–Cu/char (8 : 2) catalyst was effective in converting BO-CS into hydrogen-rich products with increased overall reforming performance, while simultaneously reducing coke deposition at elevated temperatures.

**Fig. 5:**
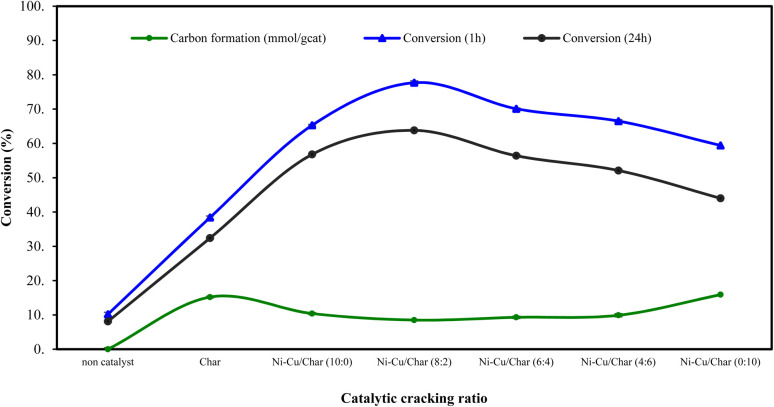
Conversion efficiency of Ni–Cu/char catalysts with different Ni–Cu ratios during pyrolysis-steam reforming.

Another test involved the long-term stability evaluation of the Ni–Cu/char (8 : 2) catalyst under combined steam and oxygen conditions at 800 °C for 72 h. The variations in conversion, hydrogen yield, and product gas distribution with time on stream are presented in Fig. 5. At the initial stage (3 h), the catalyst exhibited a high conversion efficiency of 80.5% and an H_2_ yield of 74.5%. The co-products included CO (40.6%), CO_2_ (52.6%), CH_4_ (2.7%), and a trace amount of C_2_^+^ hydrocarbons, indicating that the dominant reactions were steam reforming and the water–gas shift process. As the reaction progressed beyond 3 h, both the conversion and hydrogen yield gradually declined. At 30 h, the hydrogen yield and conversion decreased to 69.8% and 79.5%, respectively, while a slight carbon deposition of approximately 0.3% was observed, suggesting partial deactivation of the Ni–Cu/char (8 : 2) catalyst. After 72 h, the conversion further decreased to 71.2%, and the H_2_ yield dropped to 64.9%, corresponding to an overall loss of about 11.5% and 9.6%, respectively, relative to the initial values.

Throughout the 72 h test, the CO fraction slightly decreased from 40.6% to 35.3%, while CO_2_ increased from 52.6% to 58.3%, reflecting the oxidation of CO and surface carbon species in the presence of oxygen. Meanwhile, CH_4_ remained nearly constant at around 2.2–2.7%, indicating stable reforming performance and limited methanation activity. Under the optimized operating conditions at 800 °C, the first 3 h represented the period of maximum hydrogen production and conversion efficiency. Beyond this point, a gradual decline in activity was observed, mainly attributed to the progressive accumulation of carbon deposits on the catalyst surface, which hindered access to active sites and reduced overall reforming efficiency. Nevertheless, the synergistic interaction between Ni and Cu effectively enhanced reforming activity and facilitated *in situ* carbon removal through oxidative gasification. This cooperative behavior-maintained catalyst stability and sustained hydrogen production throughout the 72 h reaction period, confirming the robustness of the Ni–Cu/char (8 : 2) catalyst under prolonged steam-oxygen reforming conditions.

### 3.6 Physicochemical characterization of the catalyst

**Fig. 6:**
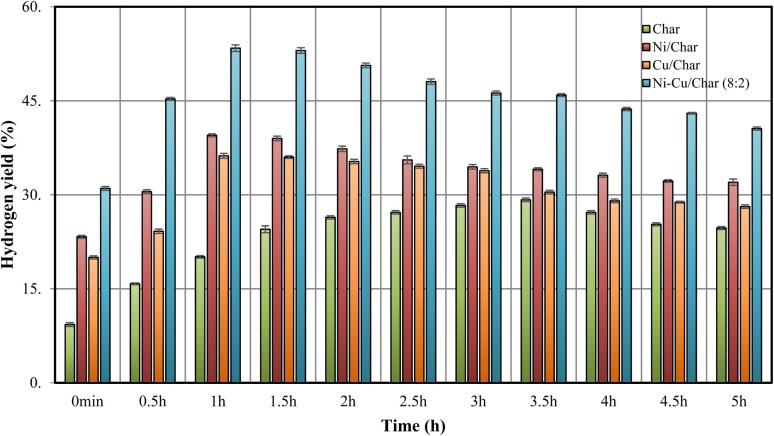
Hydrogen yield *vs.* reaction time for various catalysts in the steam reforming of bio-oil from corn stover.

The morphological and elemental analyses of the Ni–Cu/char (8 : 2) catalyst were conducted using SEM-EDX, as shown in Fig. 6(a and b).

The SEM micrographs revealed a highly porous surface structure with agglomerated and irregular carbon particles, forming a network of interconnected pores. This porous architecture provides a large specific surface area and numerous active sites that facilitate metal dispersion compared with pure char. Meanwhile, the corresponding EDX elemental mapping (Fig. 6c–e) confirmed the uniform distribution of carbon (C), nickel (Ni), and copper (Cu) across the catalyst surface. Fig. 6d and e illustrate that Ni and Cu signals were evenly dispersed, indicating that both metals were well incorporated into the carbon structure without significant phase segregation. The homogeneous dispersion of Ni and Cu suggests the possible formation of a bimetallic Ni–Cu alloy phase, which is consistent with the XRD analysis showing diffraction peaks corresponding to the alloy structure. Previous studies have also reported similar morphological characteristics and dispersion behavior of Ni and Cu^21^ observed uniformly dispersed Ni–Cu nanoparticles on biochar supports prepared *via* wet impregnation, leading to improved hydrogen yields during biomass reforming. Similarly,^22^ demonstrated that bimetallic Ni–Cu catalysts supported on nanocarbon structures exhibited smaller particle sizes and greater resistance to carbon deposition than monometallic Ni catalysts. Moreover,^23^ reported that the high dispersion of Ni and Cu on porous carbon enhanced the metal support interaction, thereby improving the structural stability of the catalyst under high-temperature operating conditions.

**Fig. 7:**
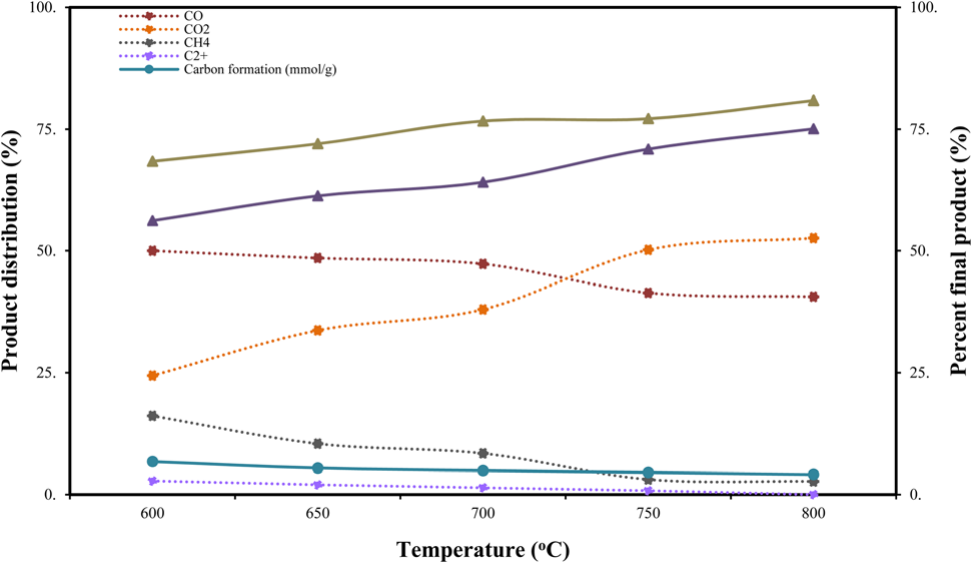
Influence of temperature (600–800 °C) on product distribution (CO, CO_2_, CH_4_, and C_2_^+^), hydrogen yield (%), feedstock conversion (%), and carbon formation (mmol g^−1^) during catalytic steam reforming of corn stover bio-oil over Ni–Cu/char (8 : 2) catalyst. An increase in temperature led to higher H_2_ yield and conversion, decreased hydrocarbon content (CH_4_, C_2_^+^), and reduced carbon formation, while CO_2_ production increased and CO slightly decreased, indicating thermodynamically favorable reforming and water–gas shift reactions.

The FTIR spectra of the Ni–Cu/carbon catalyst before and after the reaction are presented in Fig. 7. Only minor structural changes were observed between the two spectra, indicating that the chemical structure of the catalyst remained stable during the reaction. The prominent band observed around 1062–1067 cm^−1^ is attributed to the stretching vibration of C–O or Si–O bonds, suggesting the presence of oxygen-containing functional groups originating from the carbon support. A weak band near 755 cm^−1^ is assigned to the out-of-plane C–H bending of aromatic rings, indicating that aromatic carbon groups remained on the catalyst surface. After the catalytic reaction, slight changes were observed in the stretching bands of CO and C–O, which could be attributed to minor oxidation or surface reconstruction of the metallic phase during the steam reforming process. The minimal variation in transmittance intensity further confirms that the Ni–Cu/carbon catalyst maintained good structural stability and chemical integrity under the reaction conditions.

**Fig. 8:**
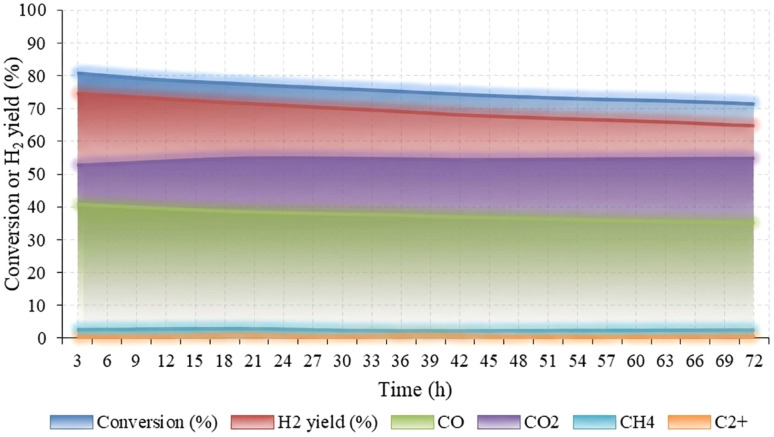
Conversion or H_2_ yield (%) and product distribution over time (h).

**Fig. 9:**
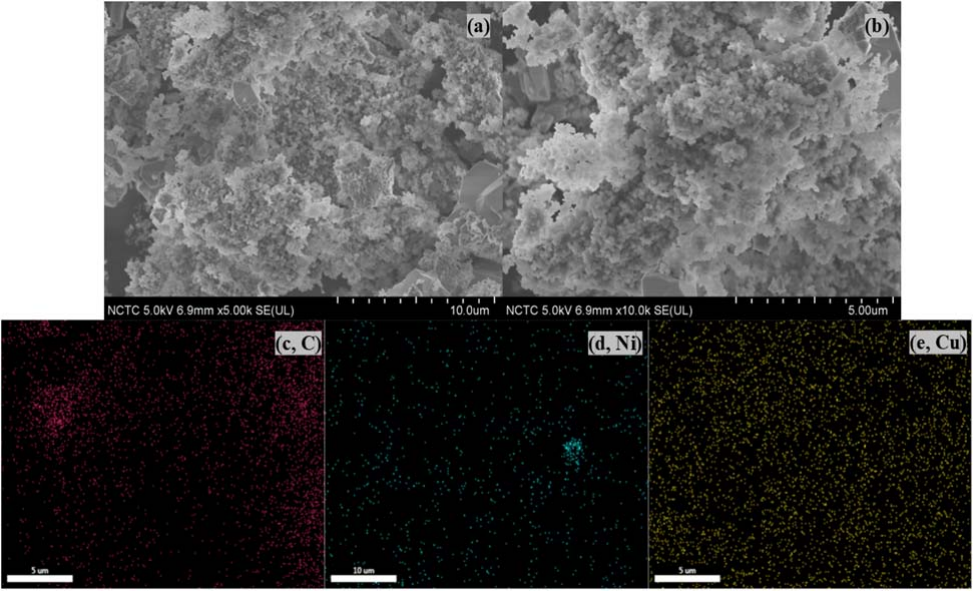
SEM (a and b) and EDX (c–e) image of Ni–Cu/char (8 : 2).

**Fig. 10:**
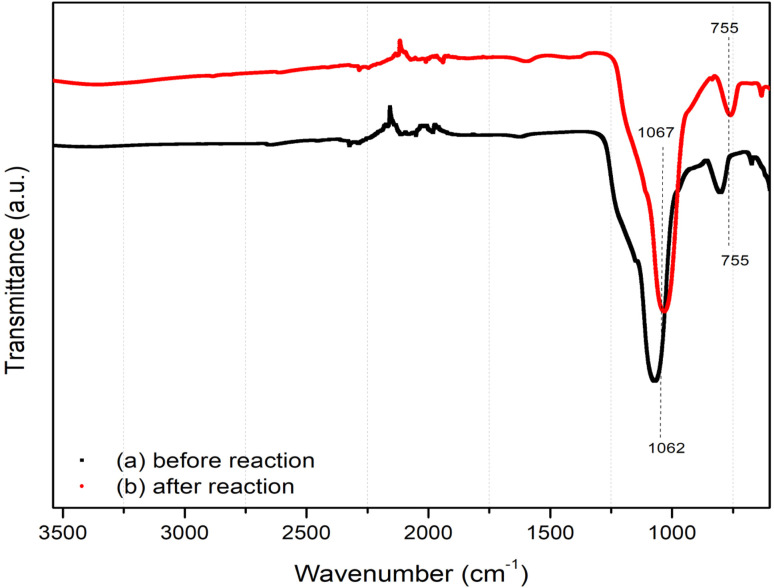
FTIR spectra of the Ni–Cu/carbon catalyst before and after reaction.

The XRD patterns of the Ni–Cu/carbon (8 : 2) catalyst before and after the reaction are presented in Fig. 8. The pattern recorded before reaction corresponds to the catalyst after calcination followed by *in situ* H_2_ reduction at 400 °C prior to catalytic testing. The results show only slight differences between the two spectra Fig. 9. Distinct diffraction peaks were observed at approximately 2*θ* values of 44° and 51°, which correspond to the (111) and (200) planes of metallic Ni, respectively, indicating the presence of crystalline Ni nanoparticles. No distinct diffraction peaks corresponding exclusively to metallic Cu were observed. This may be attributed to the relatively low Cu loading, the possible formation of Ni–Cu alloy phases with overlapping diffraction peaks, and the high dispersion or small crystallite size of Cu species, which may render their signals below the detection limit of XRD analysis (Fig. 10).

**Fig. 11:**
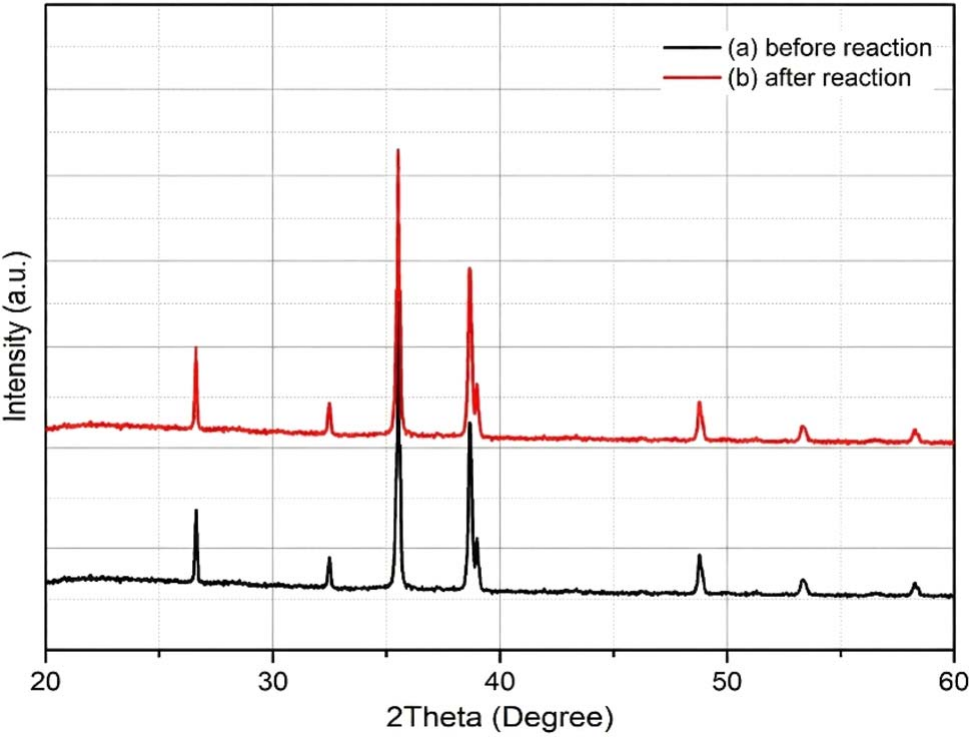
XRD Analysis of Ni–Cu/carbon catalyst before and after reaction.

No additional peaks associated with NiO or CuO phases were detected after the reaction, suggesting that the metallic phases of Ni and Cu remained stable under the reaction conditions. A slight decrease in the intensity of the Ni peaks after the reaction may be attributed to partial encapsulation of Ni nanoparticles by carbon species accumulated during the catalytic process. In addition, a small hump related to amorphous carbon structures was observed, which corresponds to the deposition of carbon (coke formation) on the catalyst surface. Previous studies have reported similar observations, indicating that Ni–Cu catalysts can preserve their metallic crystalline structure after reforming or steam reforming processes.^24^ observed the persistence of Ni–Cu alloy peaks in a Ni–Cu/CeMnO_2_ catalyst after the steam reforming of ethanol, demonstrating its high thermal stability. Similarly,^25^ and^26^ reported that Ni-based catalysts supported on carbonaceous materials exhibited only minor peak shifts without significant structural degradation of the catalyst (Fig. 11).

The textural characteristics of the char support and metal-loaded catalysts were examined using N_2_ adsorption–desorption analysis, and the results are summarized in Table 4. The untreated char exhibited a specific surface area of approximately 28–35 m^2^ g^−1^ and a total pore volume of about 0.04–0.08 cm^3^ g^−1^, indicating a moderately developed porous structure capable of accommodating metal species. Following metal impregnation, a reduction in both surface area and pore volume was observed for all catalysts, which is commonly attributed to partial pore filling and surface coverage by deposited metal particles. Among the prepared samples, the Ni–Cu/char catalyst with a Ni–Cu ratio of 8 : 2 maintained a relatively higher surface area (∼30 m^2^ g^−1^) compared with the monometallic Ni/char catalyst (∼28 m^2^ g^−1^). This observation suggests that the incorporation of a small amount of Cu promoted better dispersion of Ni species and limited excessive pore blockage. In contrast, catalysts with higher Cu contents (Ni–Cu ratios of 6 : 4, 4 : 6, and 0 : 10) exhibited a progressive decline in surface area and pore volume, reaching values as low as ∼24 m^2^ g^−1^ and ∼0.068 cm^3^ g^−1^, respectively. This decrease is likely associated with agglomeration of Cu species and increased obstruction of the pore network, which can restrict reactant diffusion and reduce accessibility to active sites. Overall, the Ni–Cu/char (8 : 2) catalyst achieved an optimal balance between surface area preservation and metal dispersion, which is consistent with its superior catalytic activity, enhanced hydrogen yield, and improved stability during the steam reforming of corn stover bio-oil.

**Table 4:** BET analysis of PCH Ni/Cu

Sample	*S*_BET_ (m^2^ g^−1^)	Total pore volume (cm^2^ g^−1^)
Char	28	0.04
Ni–Cu/char (10 : 0)	28	0.075
Ni–Cu/char (8 : 2)	30	0.072
Ni–Cu/char (6 : 4)	27	0.07
Ni–Cu/char (4 : 6)	25	0.069
Ni–Cu/char (0 : 10)	24	0.068

In addition, to clarify the structure activity relationship associated with hydrogen production, surface properties were further examined by XPS, and the results are presented in Table 5. The analysis provides a comparative evaluation of the surface composition and oxidation states of the Ni–Cu/char catalysts. The Ni 2p spectra indicate that the fraction of metallic Ni^0^ strongly depends on the Ni–Cu ratio. The Ni–Cu/char (8 : 2) catalyst exhibits the highest proportion of Ni^0^ (75.5%), which is significantly higher than that observed for the monometallic Ni/char catalyst (61.2%). This result suggests that the incorporation of Cu promotes the reduction and stabilization of metallic Ni species on the catalyst surface.

**Table 5:** Surface composition and oxidation states of Ni–Cu/char catalysts determined by XPS analysis

Catalyst	Ni^0^ (%)	Ni^2+^ (%)	Cu^0^/Cu^+^ (%)	Cu^2+^ (%)	Ni/Cu (surface ratio)
Ni–Cu/char (10 : 0)	61.2	38.7	—	—	—
Ni–Cu/char (8 : 2)	75.5	25.5	68.0	31.8	3.9
Ni–Cu/char (6 : 4)	66.4	33.6	63.5	36.5	1.8
Ni–Cu/char (4 : 6)	54.7	45.3	58.4	41.6	0.9
Ni–Cu/char (0 : 10)	—	—	49.2	50.8	—

Meanwhile, the Cu 2p spectra reveal that the 8 : 2 catalyst contains a higher fraction of reduced copper species (Cu^0^/Cu^+^, 68.0%) compared with Cu-rich catalysts. The presence of partially reduced copper species is considered beneficial for facilitating the water gas shift reaction and for suppressing carbon deposition during steam reforming. Furthermore, the surface atomic Ni/Cu ratio shows that the 8 : 2 catalyst maintains a balanced surface composition, which favors synergistic interactions between Ni and Cu. In contrast, increasing the Cu content results in a higher proportion of Cu^2+^ species and a concomitant decrease in metallic Ni fraction, which is consistent with the progressive decline in hydrogen yield and feedstock conversion observed for Cu-rich catalysts.

### 3.7 Kinetic analysis and apparent activation energy of hydrogen formation

To further elucidate the intrinsic catalytic behavior and quantitatively assess the promotional effect of Cu on Ni-based catalysts, a kinetic analysis of hydrogen formation during the steam reforming of corn stover bio-oil was conducted. Apparent activation energies (*E*_a_) were determined for char, Ni/char, Cu/char, and Ni–Cu/char (8 : 2) catalysts based on temperature-dependent hydrogen production rates measured in the range of 600–800 °C. The reaction rate of hydrogen formation (*r*_H_2__) was calculated from the molar hydrogen production normalized to catalyst mass and reaction time. Assuming that the overall reaction follows apparent first-order kinetics with respect to bio-oil-derived hydrocarbons under excess steam conditions, the Arrhenius relationship can be expressed as:
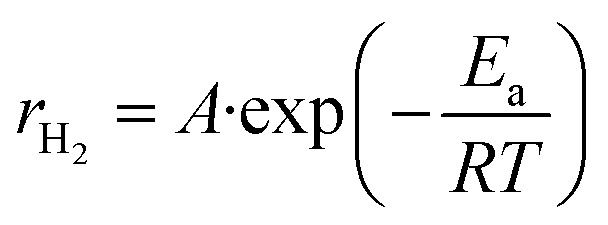

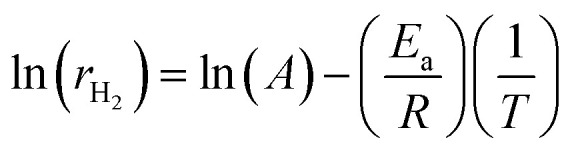
*E*_a_ = −(Slope) × *R*where *A* is the pre-exponential factor, *E*_a_ is the apparent activation energy (kJ mol^−1^), *R* is the universal gas constant, and *T* is the absolute temperature (K). Linear regression of ln(*r*_H_2__) *versus* 1/*T* was employed to extract *E*_a_ values for each catalytic system. The calculated apparent activation energies followed the order: Char > Cu/char > Ni/char > Ni–Cu/char (8 : 2) (Table 6).

**Table 6:** Apparent activation energy for H_2_ formation during steam reforming of BO-CS (600–800 °C)^a^

Catalyst	H_2_ (vol%) used at 600/700/750/800 °C	*E*_a_ (kJ mol^−1^)	ln(*A*)	*A* (mol g_cat_^−1^ s^−1^)	*R* ^2^
Non-catalytic	5/7/8/10	26.88	−6.5109	1.49 × 10^−3^	0.9942
Char	15/20/22/25	19.91	−6.3562	1.74 × 10^−3^	0.9998
Cu/char (0 : 10)	30/38/41/45	15.83	−6.2239	1.98 × 10^−3^	0.9987
Ni/char (10 : 0)	45/55/60/65	14.32	−6.0308	2.40 × 10^−3^	1.000
NiCu/char (8 : 2)	56/64/70/75	11.31	−6.2346	1.96 × 10^−3^	0.9882

a Apparent activation energies were obtained from Arrhenius plots of ln(*r*_H_2__) *versus* 1/*T* over 600–800 °C, where *r*_H_2__ was calculated from outlet H_2_ molar flow normalized by catalyst mass.

**Fig. 12:**
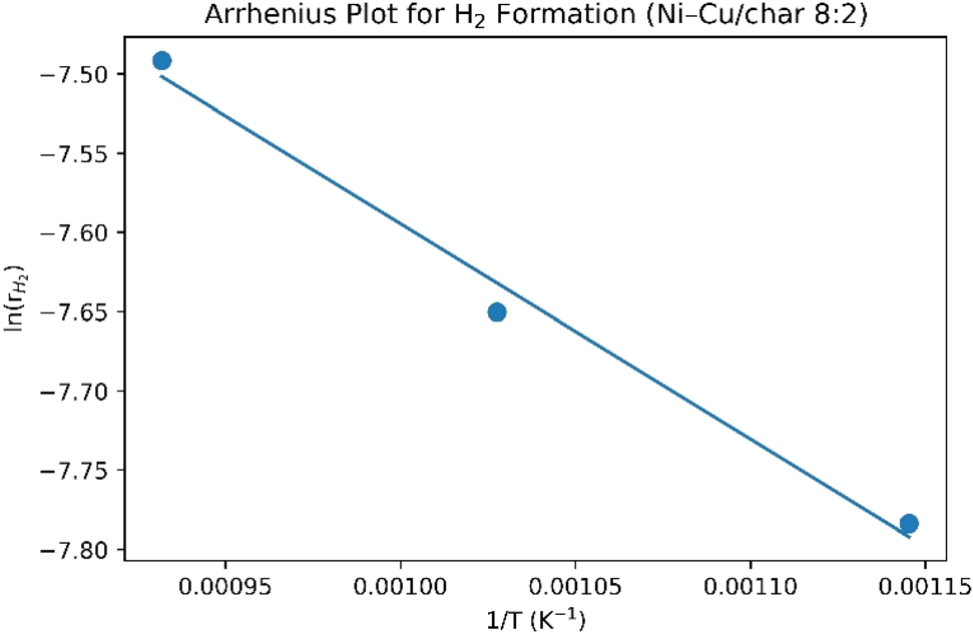
Arrhenius plot for hydrogen formation during steam reforming of corn stover bio-oil over Ni–Cu/char (8 : 2) catalyst.

It can be seen (Table 7 and Fig. 12) that the char-supported system exhibited the highest *E*_a_, indicating that hydrogen formation was predominantly governed by non-catalytic thermal cracking and limited surface-mediated reforming. The Cu/char catalyst showed a modest reduction in *E*_a_, reflecting Cu's limited intrinsic activity for C–C bond cleavage. In contrast, the monometallic Ni/char catalyst displayed a substantially lower activation energy, confirming the well-known role of Ni in facilitating hydrocarbon activation and steam reforming reactions.

**Table 7:** Temperature-dependent H_2_ molar flow and H_2_ formation rate used for Arrhenius analysis (Ni–Cu/char 8 : 2)

Temp. (°C)	*T* (K)	1/*T* (K^−1^)	H_2_ (vol.)	*F*_total_ (mol s^−1^)	*F*_H_2__ (mol s^−1^)	*r*_H_2__ (mol g_cat_^−1^ s^−1^)	ln(*r*_H_2__)
600	873.1	0.001	56	7.44 × 10^−5^	4.16 × 10^−5^	4.16 × 10^−4^	−7.783
700	973.1	0.001	64	7.44 × 10^−5^	4.76 × 10^−5^	4.76 × 10^−4^	−7.650
750	1023.1	0.001	70	7.44 × 10^−5^	5.19 × 10^−5^	5.19 × 10^−4^	−7.563
800	1073.1	0.000	75	7.44 × 10^−5^	5.58 × 10^−5^	5.58 × 10^−4^	−7.491

Notably, the Ni–Cu/char (8 : 2) catalyst exhibited the lowest apparent activation energy (Table 6), demonstrating a clear kinetic advantage over the monometallic Ni catalyst. This reduction in *E*_a_ indicates that the incorporation of Cu into the Ni lattice alters the electronic and geometric environment of Ni active sites, thereby lowering the energy barrier for hydrocarbon reforming and hydrogen generation. The synergistic interaction between Ni and Cu is attributed to enhanced surface oxygen mobility, improved water activation, and suppression of strongly bound carbonaceous intermediates that typically inhibit Ni active sites. These kinetic findings strongly support the experimental observations of higher hydrogen yield, improved feedstock conversion, and enhanced coke resistance for the Ni–Cu/char (8 : 2) catalyst. The lower activation energy confirms that the improved performance of the bimetallic catalyst is not solely thermodynamically driven but is fundamentally rooted in favorable reaction kinetics. Consequently, the Ni–Cu/char (8 : 2) catalyst enables more efficient hydrogen production at elevated temperatures while maintaining long-term catalytic stability, reinforcing its suitability for practical biomass-to-hydrogen conversion processes.

### 3.8 Carbon balance and hydrogen efficiency of BO-CS steam reforming

A quantitative carbon balance was conducted to assess both the efficiency of carbon conversion and the reliability of product quantification during the catalytic steam reforming of corn stover bio-oil. The balance was established by comparing the total carbon introduced with the bio-oil feed to the carbon recovered in gaseous products (CO, CO_2_, CH_4_, and C^2+^ hydrocarbons) as well as the carbon retained as solid deposits on the catalyst surface. Gas-phase carbon was determined from GC-measured product compositions combined with outlet molar flow rates, whereas deposited carbon was independently quantified *via* temperature-programmed oxidation (TPO) of the spent catalysts, enabling accurate identification of coke species.

The resulting carbon balance closure of 92–97% demonstrates strong agreement between inlet and outlet carbon streams, confirming the robustness of the analytical methodology. This integrated approach provides a reliable framework for evaluating carbon deposition and substantiates the conclusion that the bimetallic Ni–Cu/char catalyst effectively suppresses coke formation during steam reforming.

Under the investigated conditions (600–800 °C), the overall carbon balance closure ranged between 92–97%, indicating good agreement between inlet and outlet carbon and confirming the robustness of the experimental measurements. Minor carbon deficits can be attributed to trace losses associated with undetected heavy hydrocarbons, tar condensation, and experimental uncertainties inherent to high-temperature reforming systems. Notably, carbon balance closure improved with increasing temperature, consistent with enhanced cracking and reforming of heavy oxygenated compounds into detectable gaseous products.

Beyond the carbon balance, hydrogen efficiency was assessed to quantitatively determine how effectively the hydrogen inherently present in the bio-oil feedstock was transformed into molecular hydrogen during steam reforming. Hydrogen efficiency (*η*_H_2__) was defined as:
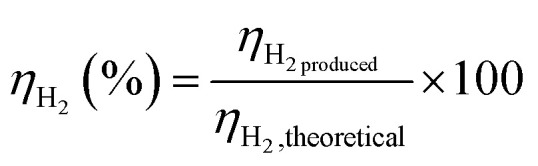
*C*_feed_ = *n*_feed_ × (mol C per mol feed)
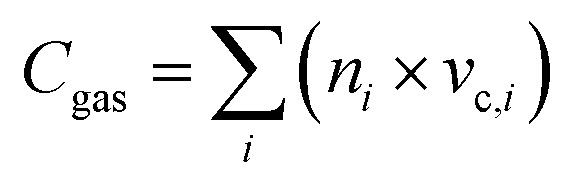
*C*_coke_ = TPO-derived deposited carbon (converted to mmol C)*C*_unacc_ = *C*_feed_ − *C*_gas_ − *C*_coke_
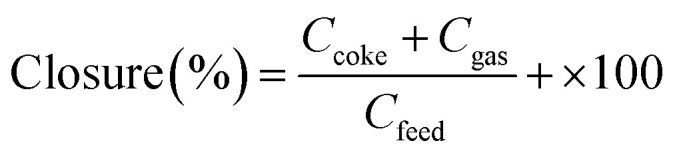
where *η*_H_2_produced_, produced represents the experimentally measured molar amount of hydrogen at the reactor outlet (*Fr*_H_2__ = *y*_H_2__*F*_total_) and *η*_H_2_,theoretical_ corresponds to the maximum hydrogen obtainable from complete reforming and water gas shift conversion of the bio-oil feed. Using bio-oil (C_10_H_8_) as a representative model compound for bio-oil aromatics, the theoretical maximum hydrogen yield is 24 mol H_2_ per mol of feed. To estimate hydrogen efficiency, the theoretical maximum hydrogen yield was calculated using naphthalene (C_10_H_8_) as a surrogate aromatic compound. This choice was intended to approximate the behavior of refractory aromatic species that commonly arise from lignin decomposition during biomass pyrolysis and are known to influence reforming pathways and coke formation. Selecting an aromatic surrogate provides a conservative basis for calculating hydrogen potential, thereby reducing the likelihood of overstating efficiency that might occur if more hydrogen-rich compounds were assumed. It should be emphasized that naphthalene was not employed as the experimental feedstock; the reforming experiments were performed using whole corn stover bio-oil in order to preserve the chemical heterogeneity characteristic of real biomass-derived liquids. Therefore, the theoretical hydrogen value is best interpreted as a stoichiometric reference for comparative evaluation of catalytic performance rather than as a precise compositional description of the bio-oil. This clarification is provided to enhance methodological transparency and to support a balanced interpretation of the hydrogen efficiency results.

The Ni–Cu/char (8 : 2) catalyst exhibited the highest hydrogen efficiency among all tested systems, reaching approximately 50–55% of the theoretical hydrogen potential at 800 °C, which is significantly higher than those obtained with char, Cu/char, or monometallic Ni/char catalysts. The improvement in hydrogen efficiency is attributed to the synergistic interaction between Ni and Cu, where Ni promotes effective C–C and C–H bond cleavage, while Cu enhances the water–gas shift reaction and suppresses carbon–metal interactions, thereby minimizing hydrogen losses associated with coke formation and hydrocarbon by-products. Overall, the combined carbon balance and hydrogen efficiency analysis confirms that the Ni–Cu/char (8 : 2) catalyst enables efficient carbon utilization and effective hydrogen recovery from whole bio-oil. These results demonstrate that the improved hydrogen yield observed for the bimetallic catalyst is not merely a consequence of favorable product distribution but reflects a genuine enhancement in carbon conversion efficiency and hydrogen generation performance, reinforcing the suitability of this catalytic system for practical biomass-to-hydrogen applications.

The integrated analysis shows that increasing temperature simultaneously improves hydrogen efficiency and carbon balance closure (Tables 8–10). At 800 °C, hydrogen efficiency reached 75% of the theoretical maximum, while 93% of the carbon in the feedstock was converted into gaseous products with minimal coke formation. These results confirm that enhanced hydrogen recovery is directly linked to improved carbon utilization and effective suppression of solid carbon deposition.

**Table 8:** Hydrogen efficiency for Ni–Cu/char (8 : 2)

Temp. (°C)	H_2_ (vol%)	*F*_total_ (mol s^−1^)	*F*_H_2__ = *y* × *F*_total_ (mol s^−1^)	Theoretical H_2_ (mol s^−1^)	H_2_ efficiency (%)
600	56	7.44 × 10^−5^	4.16 × 10^−5^	7.44 × 10^−5^	56
700	64	7.44 × 10^−5^	4.76 × 10^−5^	7.44 × 10^−5^	64
800	75	7.44 × 10^−5^	5.58 × 10^−5^	7.44 × 10^−5^	75

**Table 9:** Integrated carbon balance and hydrogen efficiency during steam reforming of corn stover bio-oil over Ni–Cu/char (8 : 2)

Temperature (°C)	a	b	c	d	e	f	g
600	56	56	100	86	6	8	92
700	64	64	100	90	5	5	95
800	75	75	100	93	4	3	97

a Measured H_2_ (vol%).

b Hydrogen efficiency (% of theoretical).

c Carbon in feed (mmol C).

d Carbon in gas (mmol C).

e Carbon as coke (mmol C).

f Unaccounted carbon (mmol C).

g Carbon balance closure (%).

**Table 10:** Carbon balance calculation for Ni–Cu/char 8 : 2

Temp. (°C)	aCarbon in feed (mmol C)	bCarbon in gas (mmol C)	cCarbon as coke (mmol C)	dUnaccounted carbon = *c* − *d* − *e* (mmol C)	eClosure = (*d* + *e*)/*c* × 100 (%)
600	100	86	6	100 − 86 − 6 = 8	(86 + 6)/100 × 100 = 92
700	100	90	5	100 − 90 − 5 = 5	(90 + 5)/100 × 100 = 95
800	100	93	4	100 − 93 − 4 = 3	(93 + 4)/100 × 100 = 97

a Carbon in feed (mmol C) represents the total amount of carbon introduced with the bio-oil feed over the selected experimental basis. In this work, the inlet carbon was normalized to 100 mmol C to facilitate comparison across temperatures.

b Carbon in gas (mmol C) is the total carbon recovered in gaseous products, calculated from GC-measured outlet compositions and outlet molar flow rates by summing the carbon contributions of CO, CO_2_, CH_4_, and C_2_^+^ hydrocarbons.

c Carbon as coke (mmol C) corresponds to solid carbon deposited on the spent catalyst, quantified independently by temperature-programmed oxidation (TPO) of the used catalysts and reported as mmol of carbon.

d Unaccounted carbon (mmol C) was determined by difference: (*f* = *c* − *d* − *e*). This term represents minor carbon losses and uncertainties, including undetected heavy hydrocarbons/tars, condensation losses, and experimental measurement uncertainty.

e Carbon balance closure (%) was calculated as *g* = ((*d* + *e*)/*c*) × 100, indicating the fraction of inlet carbon recovered as gas plus coke.

### 3.9 Significance and novelty

This study addresses key challenges in renewable hydrogen production by developing a robust catalytic system for the steam reforming of whole bio-oil derived from corn stover, a realistic and chemically complex lignocellulosic feedstock. Unlike many previous studies relying on single model compounds, this work evaluates catalyst performance under conditions that closely reflect practical biorefinery operation, thereby enhancing its industrial relevance. The results demonstrate that char-supported Ni–Cu bimetallic catalysts, particularly at an optimized Ni–Cu ratio of 8 : 2, achieve high hydrogen yields (>50%) while significantly suppressing coke formation, overcoming a major limitation of conventional Ni-based reforming catalysts. The novelty of this work lies in (i) the use of whole bio-oil instead of model oxygenates, (ii) the identification of an optimal Ni–Cu synergy on a char support, and (iii) the mechanistic clarification of distinct Ni and Cu roles in reforming and water–gas shift reactions. In addition, long-term stability tests (up to 72 h) confirm sustained catalytic activity under severe steam–oxygen conditions. Collectively, this study provides new mechanistic and practical insights into char-supported bimetallic catalysts and advances sustainable, circular pathways for biomass-to-hydrogen conversion.

The comparative analysis in Table S1 highlights clear performance advantages of the present Ni–Cu/char (8 : 2) catalyst over recently reported Ni-based systems for biomass-derived bio-oil steam reforming. Most prior studies have focused on simplified model compounds such as acetic acid, ethanol, or phenol, which do not fully capture the chemical complexity and coking tendency of real bio-oils. Even when real bio-oil feeds were employed, hydrogen yields typically remained below 50% and were often accompanied by rapid catalyst deactivation or significant coke formation. In contrast, the Ni–Cu/char (8 : 2) catalyst developed in this work achieved a hydrogen yield exceeding 52% at 800 °C while maintaining low carbon deposition (∼4 mmol g^−1^), demonstrating superior resistance to coking under severe reforming conditions. The enhanced performance is attributed to the synergistic interaction between Ni and Cu, where Ni provides high reforming activity *via* C–C and C–H bond cleavage, while Cu promotes the water–gas shift reaction and weakens carbon–metal interactions, thereby suppressing coke formation. Furthermore, the use of char as a support contributes to improved metal dispersion and aligns with circular biorefinery principles. Collectively, this comparison confirms that the present catalytic system represents a meaningful advancement over existing literature in terms of realism, efficiency, and long-term stability.

## 4. Conclusion

This work demonstrates an efficient and sustainable route for hydrogen production from whole bio-oil derived from corn stover through an integrated fast pyrolysis–catalytic steam reforming process. Corn stover was confirmed to be a suitable lignocellulosic feedstock, producing a high yield of oxygenated bio-oil under optimized fast pyrolysis conditions, which is well suited for downstream reforming applications. The catalytic steam reforming results clearly show that catalyst composition plays a decisive role in hydrogen yield, feedstock conversion, and coke resistance. Among all tested systems, the char-supported Ni–Cu bimetallic catalyst exhibited superior performance compared with non-catalytic, char-only, and monometallic catalysts. In particular, the Ni–Cu/char catalyst with an optimized Ni–Cu ratio of 8 : 2 achieved the highest hydrogen yield (∼52–53%) and feedstock conversion (∼77–78%) at 800 °C, while maintaining low carbon deposition (∼4 mmol g^−1^). The enhanced catalytic performance is attributed to the synergistic interaction between Ni and Cu, where Ni provides high reforming activity *via* effective C–C and C–H bond cleavage, while Cu promotes the water–gas shift reaction and weakens carbon–metal interactions, thereby suppressing coke formation and improving catalyst stability. Temperature and time-on-stream investigations further confirmed that higher operating temperatures favor hydrogen production, hydrocarbon cracking, and *in situ* coke gasification. Long-term stability tests up to 72 h demonstrated that the Ni–Cu/char (8 : 2) catalyst retains acceptable activity under severe steam–oxygen reforming conditions. Comprehensive physicochemical characterization confirmed uniform metal dispersion, structural robustness, and preserved porosity of the Ni–Cu/char catalyst after reaction. Collectively, these findings provide both mechanistic insight and practical evidence that char-supported Ni–Cu bimetallic catalysts represent a promising, circular, and scalable approach for converting agricultural residues into hydrogen-rich gas. This study therefore contributes to advancing integrated biomass-to-hydrogen pathways and supports the development of sustainable biorefinery systems based on real bio-oil feedstocks.

## Author contributions

Surachai Wongcharee: writing – review & editing, writing – original draft, visualization, validation, formal analysis. Nopparat Suriyachai and Torpong Kreetachat: writing – review & editing, writing – original draft, resources, methodology, funding acquisition, conceptualization. Methawee Nukunudompanich: writing – review & editing, writing – original draft. Supachai Jadsadajerm: resources, project administration. Saksit Imman: writing – review & editing, writing – original draft, methodology, formal analysis, funding acquisition, conceptualization.

## Conflicts of interest

The authors declare that they have no known competing financial interests or personal relationships that could have appeared to influence the work reported in this paper.

## Supplementary Material

RA-016-D6RA00271D-s001

## Data Availability

Data will be made available on request. Supplementary information (SI) is available. See DOI: https://doi.org/10.1039/d6ra00271d.
