# Supplementary material for: Synergistic Ni–Cu/char bimetallic catalysts for enhanced hydrogen production from corn stover bio-oil via steam reforming
Source: RSC Adv. 2026 Mar 19;16(17):15626–39. doi: 10.1039/d6ra00271d (PMC13001720; doi:10.1039/d6ra00271d)
Supplement: RA-016-D6RA00271D-s001 [file RA-016-D6RA00271D-s001.pdf]

Supplementary Information (SI) for RSC Advances.
This journal is © The Royal Society of Chemistry 2026

Supplementary file

Table S1. Comparison of hydrogen production performance from biomass-derived bio-oil steam reforming using Ni-based catalysts (recent literature)

Feedstock / Model compound	Catalyst system	Support	Temp. (°C)	H_2 yield / performance*	Coke formation	Key limitations	Ref.
Acetic acid (model)	Ni/ZrO_2	ZrO_2	700–800	H_2 yield ≈ 40–45%	Moderate	Model compound; limited representativeness	Li et al., 2012
Ethanol (model)	Ni–Cu/MgAl	Mixed oxide	650–750	H_2 selectivity ↑ (Cu-promoted WGS)	Reduced vs Ni	Not real bio-oil; alcohol only	Yu et al., 2019
Phenol (model)	Ni/CeO_2	CeO_2	700	Stable H_2, good conversion	Moderate	Catalyst cost; oxide sintering	Silva et al., 2022
Bio-oil model mixture	Ni/Al_2O_3–MgO	Al_2O_3	800	H_2 ≈ 45–50%	High without Mg	Coke still significant	Jin et al., 2021
Real pine bio-oil	Ni/$CeMnO_2$	Mixed oxide	750–800	H_2 ≈ 48%	Moderate	Short time-on-stream	Sohrabi et al., 2021
Biomass oxygenates	Ni/C (biochar)	Carbon	750	H_2-rich syngas	Lower than oxide	Monometallic Ni deactivation	Gai et al., 2019
Corn stover bio-oil (whole)	Ni–Cu/char (8:2)	Char	800	H_2 yield ≈ 52–53%	Low (≈4 mmol g^{-1})	—	This work
